# Type 1 Innate Lymphoid Cells Limit the Antitumoral Immune Response

**DOI:** 10.3389/fimmu.2021.768989

**Published:** 2021-11-16

**Authors:** Margaux Vienne, Marion Etiennot, Bertrand Escalière, Justine Galluso, Lionel Spinelli, Sophie Guia, Aurore Fenis, Eric Vivier, Yann M. Kerdiles

**Affiliations:** ^1^ Aix-Marseille Univ, Centre National de la Recherche Scientifique (CNRS), Institut National de la Santé et de la Recherche Médicale (INSERM), Centre d’Immunologie de Marseille-Luminy (CIML), Marseille, France; ^2^ Innate Pharma, Marseille, France; ^3^ APHM, Hôpital de la Timone, Marseille-Immunopôle, Marseille, France

**Keywords:** innate, innate lymphoid cells (ILCs), antitumoral immunity, NK cells, ILC1

## Abstract

Natural killer (NK) cells are known to be able to kill established tumor cell lines, but important caveats remain regarding their roles in the detection and elimination of developing primary tumors. Using a genetic model of selective ILC1 and NK cell deficiency, we showed that these cells were dispensable for tumor immunosurveillance and immunoediting in the MCA-induced carcinogenesis model. However, we were able to generate primary cell lines derived from MCA-induced tumors with graded sensitivity to NK1.1^+^ cells (including NK cells and ILC1). This differential sensitivity was associated neither with a modulation of intratumoral NK cell frequency, nor the capacity of tumor cells to activate NK cells. Instead, ILC1 infiltration into the tumor was found to be a critical determinant of NK1.1^+^ cell-dependent tumor growth. Finally, bulk tumor RNAseq analysis identified a gene expression signature associated with tumor sensitivity to NK1.1^+^ cells. ILC1 therefore appear to play an active role in inhibiting the antitumoral immune response, prompting to evaluate the differential tumor infiltration of ILC1 and NK cells in patients to optimize the harnessing of immunity in cancer therapies.

## Introduction

Immune deficiencies in humans and mice are often associated with higher rates of spontaneous neoplastic diseases, highlighting the role of immune cells in continual protection of the body against tumor development (1, 2). T lymphocytes have been shown to play a crucial role in this protection (3), but important caveats remain concerning innate immune system involvement. Nevertheless, despite an absence of formal proof, there are several lines of evidence suggesting an important role for NKp46^+^ innate lymphoid cells (ILCs), including NK cells and ILC1.

ILCs are the most recently discovered group of immune cells. ILCs do not express rearranged antigen-specific receptors, but can react promptly to a wide range of microbial and inflammatory signals (4). They include two closely related subsets: ILC1 and NK cells. The cells of both these subsets are characterized by the expression of the lineage-defining transcription factor T-bet and the production of interferon (IFN)-γ upon activation. However, NK cells are cytotoxic circulating cells, the maintenance of which depends on the Eomes transcription factor, whereas ILC1 are tissue-resident cells that do not express Eomes and have low levels of cytotoxicity. The precise characterization of the phenotype of human ILC1 remains in progress, and may depend on the tissue analyzed. In mice, both NK cells and ILC1 have been shown to express the lineage-defining markers NK1.1 and NKp46, and classical NK cell markers, such as NKG2D, CD94/NKG2A and CD122 (5). These phenotypic similarities made it difficult to distinguish these two types of cells until recently, and raise important questions about their functions. Indeed, studies on NK cells use experimental strategies that also target ILC1, such as *in vivo* depletion using anti-NK1.1 or anti-asialoGM1 antibodies. Retrospectively, this raises questions about the relative contributions of these two cell types to biological functions previously attributed to NK cells.

NK cells were initially discovered on the basis of their ability to detect and kill diverse tumor cell lines of various origins both *in vitro* and *in vivo*. This property has been studied in detail, and seminal *in vivo* studies have also shown that the anti-tumor function of NK cells leads to T-cell priming and protection against subsequent tumor challenges (6–9). Nevertheless, the role of NK cells in cancer immunosurveillance — the continual detection and elimination of neotransformed cells — remains ill defined. In humans, there is still little evidence to support a role for NK cells in the antitumoral responses directed against primary solid tumors. Nonetheless, NK cell infiltration in tumor tissues has been associated with a better disease prognosis in several cancer types such as clear cell renal cell carcinomas and colorectal cancers (10). By contrast, low levels of circulating NK cells, weak cytotoxic activity and the impaired expression of NK cell activation receptors have been correlated with tumor progression or relapse or the development of metastasis (10). However, no increase in cancer incidence has been reported for the few cases of NK cell deficiencies identified to date (11). In mice, treatments with anti-NK1.1 or anti-asialoGM1 antibodies leading to NK cell depletion, are associated with an increase in sarcoma formation upon injection of the carcinogen 3-methylcholantrene (MCA) *in vivo* (12, 13). In addition, mice lacking activation receptors or cytotoxic molecules expressed by NK cells, such as TRAIL, NKG2D or perforin, also display higher rates of spontaneous tumor formation and sensitivity to carcinogen-induced tumor formation (14–16).

As a corollary of cancer immunosurveillance, cancer progression has been shown to be largely dependent on immunoediting, a process whereby the selective pressure induced by the immune system leads to the selective outgrowth of tumor cell clones capable of escaping immune-mediated control (3). Indeed, primary cell lines derived from tumors grown in an immunosufficient environment are poorly immunogenic and display progressive growth when transferred into wild-type (WT) syngeneic mice. Conversely, cell lines derived from tumors developing in immunodeficient mice (unedited) are often highly immunogenic and rejected after transfer into syngeneic WT mice (3). Consistent with this process, Myc-driven lymphomas from NKG2D–deficient mice or MCA-induced tumors from NKp46-deficient mice display stronger expression of NKG2D and NKp46 ligands, respectively, than tumors isolated from WT mice (15, 17).

We used a specific model of NKp46^+^ cell deficiency to investigate the role of NK cells and ILC1 in the immunosurveillance of solid tumors. Surprisingly, NKp46-expressing cells were found not to play a crucial role in tumor immunosurveillance and immunoediting. However, using a library of MCA-induced tumors with graded sensitivity to NK1.1^+^ cells, we found that the infiltration of ILC1 into the tumor was associated with a limited NK1.1^+^ cell-mediated antitumoral response, even fostering tumor growth. In addition, RNA sequencing experiments on this library of MCA-induced tumor cells defined a core transcriptomic signature associated with tumor sensitivity to NK1.1^+^ cells *in vivo*.

## Results

### NKp46^+^ ILCs Are Dispensable for Tumor Immunosurveillance in the MCA-Induced Carcinogenesis Model

**Figure 1:**
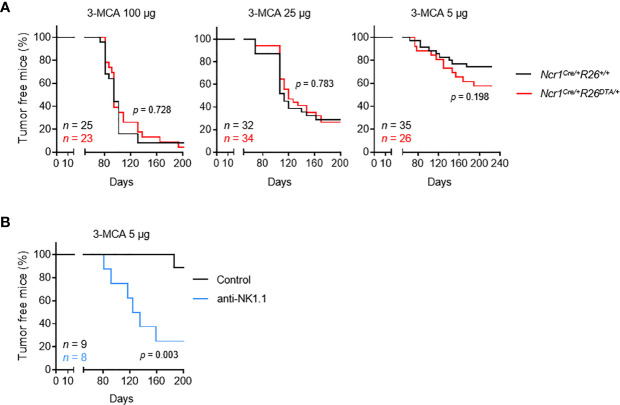
NKp46^+^ ILCs are dispensable for the immunosurveillance of MCA-induced tumors. Mice of the indicated genotype **(A)** or intravenously injected with PK136 antibody to deplete NK1.1^+^ cells **(B)** received a single subcutaneous injection of MCA at the indicated doses. Sarcoma formation was weekly monitored for 200 days. Tumor-bearing mice were defined as mice harboring masses increasing in size for at least two consecutive measurements. The *p*-values for LogRank tests are indicated.

The *Ncr1^iCre^* mouse model targets all three populations of NKp46^+^ ILCs: NK cells, ILC1 and NCR^+^ ILC3 (18). However, unlike NK cells and ILC1, which are found in the blood and diverse peripheral tissues, the NCR^+^ ILC3 cell population is found predominantly in mucosal tissues. We studied the role of these cells in tumor immunosurveillance, by monitoring the incidence of tumor formation upon MCA injection in immunologically intact mice and NKp46^+^ ILC-deficient mice (*Ncr1^Cre/+^R26^+/+^* and *Ncr1^Cre^R26^DTA/+^*, respectively) (**Figure S1A**). Cohorts of *Ncr1^Cre/+^R26^+/+^* and *Ncr1^Cre/+^R26^DTA/+^* mice received a single subcutaneous injection of 5, 25 or 100 µg of the carcinogen MCA, and tumor onset, growth and incidence were monitored over a period of 200 days **(** **Figure 1A** **)**. As expected, we observed a dose-dependent tumor incidence in both mouse strains. However, no differences were found between the two mouse genotypes for the two highest treatment doses. Tumor incidence was consistently higher in NKp46^+^ ILC-deficient mice treated with the lowest dose of MCA, although this difference was not statistically significant. These results contrast with those of previous studies reporting significantly higher rates of MCA-induced tumor formation upon treatment with anti-NK1.1 or anti-asialoGM1 depleting antibodies (12). However, given that both these types of antibodies can target cell populations other than NK cells, our results also challenge the view that only NK cells are involved in tumor immunosurveillance.

As a means of testing this hypothesis and excluding a possible bias in our experiments, we treated C57BL/6 mice with anti-NK1.1 depleting antibody targeting NKp46^+^ cells and NKT cells, which have been implicated in tumor immunosurveillance **(** **Figure S1B** **)**. This treatment significantly increased tumor formation after MCA injection **(** **Figure 1B** **)**. Overall, these results show that, in immunocompetent settings, NK cells and/or ILC1 are dispensable for tumor immunosurveillance, possibly due to functional redundancy with NKp46^-^ NK1.1^+^ cells, such as NKT cells.

### MCA-Induced Primary Fibrosarcoma Cell Lines Exhibit Various Degrees of Susceptibility to NK1.1^+^ Cells

In addition to limiting tumor formation, the selective pressure imposed by the immune system leads to the selective outgrowth of tumors with edited immunogenicity. Although the absence of NKp46^+^ cells does not alter the kinetics or incidence of tumor formation, we tested whether tumors which are growing in *Ncr1^Cre/+^R26^DTA/+^* mice were selectively susceptible to NK cells and ILC1 in WT mice.

**Figure 2:**
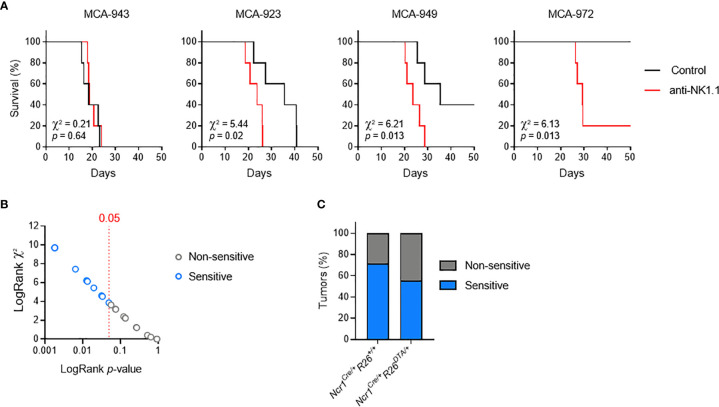
MCA-induced primary tumor cell lines with different susceptibilities to NK1.1^+^ cells. Groups of 5 C57BL/6 mice were transplanted subcutaneously with MCA-induced primary tumor cell lines, with and without treatment with a depleting anti-NK1.1 antibody. **(A)** Representative Kaplan-Meier curves. Mice were killed when the tumors reached a volume of 1,000 mm^3^. The results of LogRank tests are shown. **(B)** *p*-values and χ² of the LogRank tests associated with the survival curves of each of the primary tumor cell lines tested. One dot corresponds to one cell line. **(C)** Sensitivity status of the MCA-induced primary tumor cell lines as a function of the genotype of the mice receiving the MCA injection and developing the primary tumor.

We addressed this question by deriving primary tumor cell lines from individual MCA-induced tumors, and carrying out a large-scale *in vivo* screen to determine their sensitivity to NK cells and/or ILC1. We orthotopically transplanted MCA-induced primary tumors into immunocompetent mice, with or without anti-NK1.1 antibody treatment to deplete both NKT and NKp46^+^ cells and prevent compensatory mechanisms. By comparing the patterns of tumor growth between conditions, we identified several possible consequences of NK1.1^+^ cell depletion, ranging from no effect to increases in the incidence, growth rate of tumors, or earlier tumor onset **(** **Figure 2A** **)**. Interestingly, in all the cell lines tested, the continuous and linear relationship between χ^2^ and the *p*-values obtained in LogRank tests indicated a broad and graded reactivity to NK1.1^+^ cells **(** **Figure 2B** **)**. We used a cutoff of *p* = 0.05 for the stratification of tumor lines sensitive and non-sensitive to NK1.1^+^ cells. We tested 25 randomly selected cell lines, and found that 15 were associated with significantly lower rates of survival upon NK1.1^+^ cell depletion and were classified as NK1.1^+^ cell-sensitive **(** **Figures 2B**, **S2** **)**. Furthermore, discrimination on the basis of the original genotype revealed no difference in the overall incidence of NK1.1^+^ cell-sensitive tumors originating from WT mice and NKp46^+^ cell-deficient mice **(** **Figure 2C** **)**.

These results indicate that the immunogenicity of MCA-induced tumor cell lines is independent of NKp46^+^ NK1.1^+^ cells during primary tumor development. Thus, consistent with a dispensable role in tumor immunosurveillance in immunocompetent settings, NKp46^+^ cells alone do not impose sufficient selective pressure to contribute to tumor immunoediting. Nevertheless, this finding provided us with crucial biological tools to investigate the molecular mechanisms regulating the sensitivity of primary tumors to NK1.1^+^ cells *in vivo*.

### Tumor Cell Sensitivity Is Not Dependent on the Intratumoral Frequency of NKp46^+^ Cells

**Figure 3:**
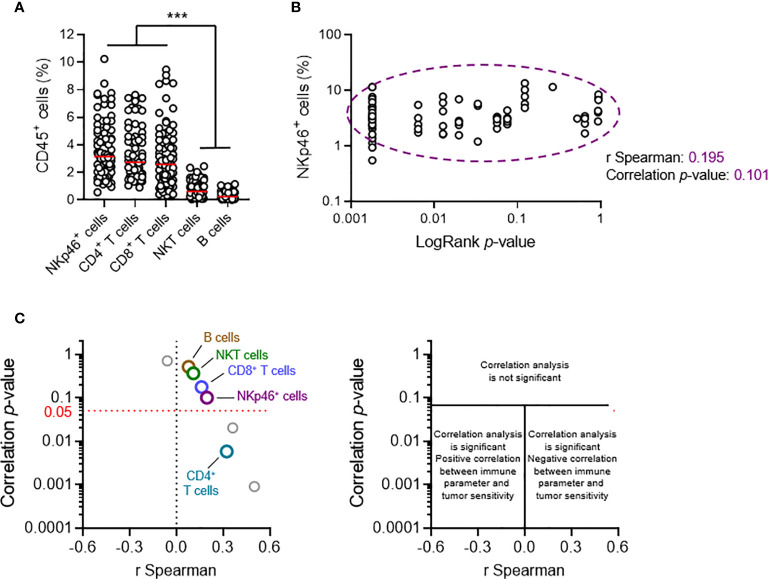
Tumor cell sensitivity is not dependent on the intratumoral frequency of NKp46^+^ cells. Groups of 5 C57BL/6 mice were transplanted subcutaneously with 25 MCA-induced primary tumor cell lines. Tumors reaching a volume of 1,000 mm^3^ were resected and tumor-infiltrating lymphocytes (TILs) were analyzed by flow cytometry. **(A, B)** Each dot represents one individual tumor from one mouse. **(A)** Frequencies of the diverse TIL populations among total CD45^+^ tumor-infiltrating cells (*n* ≥ 68 per group, ANOVA test; ***p < 0.005). Red horizontal bar represents the median. **(B)** Spearman’s correlation analysis between the frequency of tumor-infiltrating NKp46^+^ cells and the *p*-values of the LogRank tests (from **Figure 2B**) associated with each of the tumor cell lines tested (*n* = 72). **(C)** Correlation analyses, as in **(B)**, were performed for each of the parameters of tumor immune infiltration quantified. The results of Spearman’s correlation analysis are compiled in the volcano plot (correlation *p*-values and Spearman’s correlation coefficients).

Our previous results suggested that NKp46^+^ and NKT cells play redundant roles in primary tumor development. Nonetheless, a phenotypic analysis of transplanted primary tumors indicated that NKp46^+^ cells were the major infiltrating lymphocyte population, together with CD4^+^ and CD8^+^ T cells; NKT cells were also present at low levels, and B cells were barely detectable **(** **Figure 3A** **)**.

We characterized the immunological basis of this differential sensitivity, by systematically performing a broad qualitative and quantitative flow cytometry analysis of tumor infiltrating lymphocytes (TILs), and Spearman correlation analyses. We made use of the LogRank test results for survival experiments performed for each cell line. The *p*-value indicates the robustness of this difference, and the χ^2^ value indicates the effect size. The χ^2^ and *p*-values were directly proportional **(** **Figure 2B** **)**. We therefore considered using the *p*-value (*LogRank p*) as a metric describing both the extent and robustness of sensitivity to NK1.1^+^ cells for each tumor. We thus calculated Spearman’s correlation coefficients and their associated *p*-values, for the correlations between *LogRank p* and each of the phenotypic parameters measured in our screening **(** **Figures 3B, C** **)**.

At population level, the frequency of NKT cells among CD45^+^ TILs was not correlated with tumor sensitivity, arguing against a role for NKT cells in the NK1.1^+^ cell-dependent antitumoral immune response against transplanted primary tumors. We also found no correlation with the frequencies of intratumoral B cells and CD8^+^ T cells. However, there was a significant, but weak correlation with the frequency of CD4^+^ T cells. Strikingly, these data revealed that the intratumoral frequency of NKp46^+^ cells was not directly correlated with the sensitivity of the tumor to NK1.1^+^ cells **(** **Figures 3B, C** **)**.

### Tumor Cell Sensitivity Is Not Dependent on the Potential to Trigger NK Cell Activation

**Figure 4:**
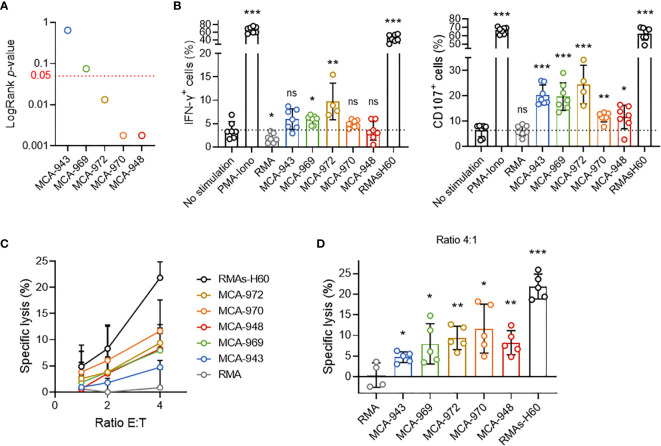
Tumor cell sensitivity is not dependent on the potential to trigger NK cell activation. **(A)** LogRank test *p*-values for MCA-tumors 948, 970, 972, 969 and 943. **(B)** IFN-γ production and CD107a expression by NK cells after 4 hours of coculture with the indicated target cells. Pooled results of at least 2 experiments per tumor cell line with each dot representing one biological replicate. **(C)** Cytotoxicity assays assessing ^51^Cr release after 4 hours of coculture of NK cells and the indicated target cells at the indicated ratios. Pooled results of at least 2 experiments per tumor cell line with at least 2 biological replicates per experiment. **(D)** Details of the cytotoxicity assays of (panel **C**) assessing ^51^Cr release after 4 hours of coculture of NK cells and the indicated target cells at 4:1 ratio. Pooled results of at least 2 experiments per tumor cell line with each dot representing one biological replicate.(mean ± SD; Student’s *t* tests (comparison with no stimulation **(B)** or RMA **(C)** conditions); *p < 0.05; **p < 0.01; ***p < 0.005; ns, not significant).

In this context, we first tested whether the differential sensitivity of primary tumor cell lines to NK1.1^+^ cells *in vivo* could stem from a differential propensity to trigger NK cell activation. We selected five representative primary tumor cell lines differing in their *in vivo* sensitivity and assessed their potential to activate NK cells **(** **Figure 4A** **)**. *In vitro* coculture with NK cells revealed the induction of very little, if any IFN-γ secretion by NK cells, but degranulation was triggered in all the cell lines tested **(** **Figure 4B** **)**. Consistent with this result, each cell line was killed by NK cells in ^51^Cr cytotoxicity assays **(** **Figures 4C, D** **)**. Nonetheless, we found no direct correlation between the extent of NK cell activation and the respective *LogRank p*-values of each cell line in either set of experiments.

Thus, although these results showed that MCA-induced primary tumor cell lines could activate NK cells, they also ruled out the magnitude of this interaction as a critical factor determining overall NK1.1^+^ cell sensitivity *in vivo*.

### Intratumoral ILC1 Play a Negative Role in the NK1.1^+^ Cell-Mediated Antitumoral Immune Response

**Figure 5:**
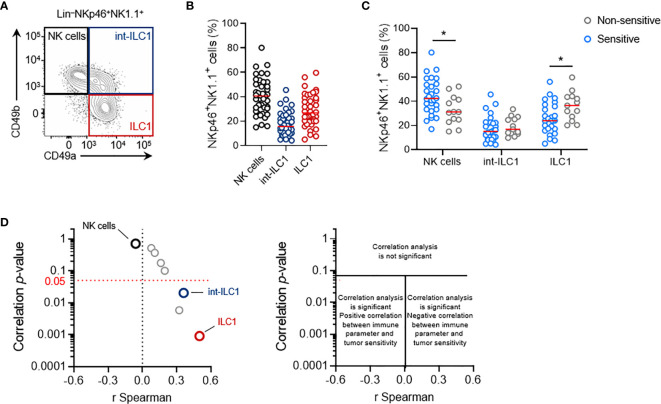
Intratumoral ILC1 play a negative role in the NK1.1^+^ cell-mediated antitumoral immune response. **(A)** Representative phenotypes and **(B)** frequencies of each subpopulation of NKp46^+^ NK1.1^+^ tumor-infiltrating cells: NK cells (CD49a^-^CD49b^+^, black), ILC1 (CD49a^+^CD49b^-^, red) and int-ILC1 (CD49a^+^CD49b^+^, dark blue). **(C)** Frequencies of tumor-infiltrating NKp46^+^ cell subpopulations stratified according to tumor sensitivity to NK1.1^+^ cells. **(B, C)** Each dot represents one individual tumor from one mouse (*n* = 40 mice per group **(B)** and *n* ≥ 13 mice per group **(C)**. Red horizontal bar represents the median (Student’s *t* tests; *p < 0.05). **(D)** Volcano plot of the correlation analysis, as in **Figure 3C**.

As previously reported (19), a close examination of NKp46^+^ TILs revealed that they consisted of a heterogeneous population of both NK cells (CD49b^+^CD49a^–^) and ILC1 (CD49a^+^CD49b^–^), together with cells expressing phenotypic markers of both populations (CD49a^+^CD49b^+^) **(** **Figures 5A, B** **)**. We therefore analyzed whether the relative proportions of these populations were linked to the magnitude of the NK1.1^+^-dependent antitumoral immune response.

Stratification of these data on the basis of the sensitivity status of each tumor cell line (cutoff set at *LogRank p* = 0.05) revealed that non-sensitive NKp46^+^ NK1.1^+^ tumor-infiltrating cells had a significantly lower frequency of NK cells, counter balanced by a larger population of ILC1 **(** **Figure 5C** **)**. This result suggests that the balance between NK cells and ILC1 in the tumor may regulate tumor growth. Interestingly, Spearman’s correlation analysis indicated that the intratumoral frequency of NK cells was not directly correlated with the degree of sensitivity to NK1.1^+^ cells of the tumors **(** **Figure 5D** **)**. However, a significant correlation was found with the level of int-ILC1 and ILC1 cell infiltration into the tumor: the highest levels of tumor infiltration were associated with the highest LogRank *p*-values, and, thus, with the lowest tumor sensitivity to NK1.1^+^ cells. Intratumoral ILC1 frequency was the parameter for which the strongest and most robust correlation was observed in our screen **(** **Figure 5D** **)**. Hence, the degree of ILC1 infiltration into the tumor appears to be a critical parameter controlling overall tumor sensitivity to NK1.1^+^ cells *in vivo*. 

Consistent with these results we also observed that the NKp46^+^ NK1.1^+^ cell infiltrates in the NK cell-sensitive B16F10 tumors consisted exclusively of NK cells **(** **Figure S3A** **)**. By contrast, the NKp46^+^ cells found in MC38 tumors included significant populations of int-ILC1 and ILC1, and the depletion of NKp46^+^ cells in *NKp46^Cre/+^R26^DTA/+^* mice was without consequence, and that of NK1.1^+^ cells in C57BL/6 mice even limited tumor growth **(** **Figures S3B, C** **)**.

These results showed that lower levels of NK cell infiltration into the tumor were not associated with a lower sensitivity of transplantable primary tumors to NK1.1^+^ cells *in vivo*. Instead, the association between the frequency of intratumoral ILC1 and tumor sensitivity to NK1.1^+^ cells suggests that intratumoral ILC1 may limit the NK1.1^+^ cell-dependent antitumoral response, or even foster tumor growth.

### Transcriptomic Profiling of Tumors With And Without Sensitivity to NK1.1^+^ Cells

Finally, we reasoned that comparing the transcriptional profiles of sensitive and non-sensitive tumors would enable us to decipher the molecular mechanisms involved in the recognition of tumor cells by NK1.1^+^ cells or tumor cell escape from these cells. We performed RNAseq analyses on 30 tumors originating from the five previously selected MCA-induced tumor cell lines with different sensitivities to NK1.1^+^ cells **(** **Figure 4A** **)**.

**Figure 6:**
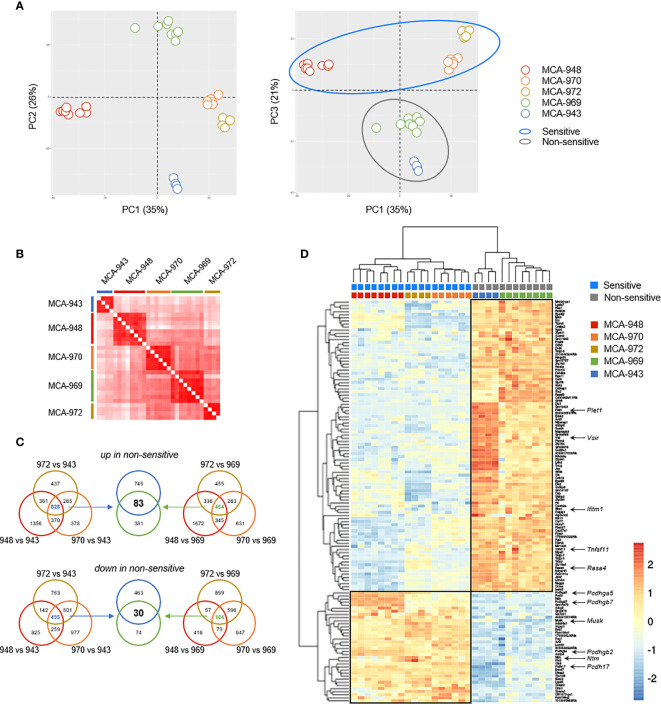
Tumor cell analysis by RNAseq. **(A)** Principal component analysis (PCA) of 30 tumors originating from 5 parental MCA-induced primary tumor cells subjected to RNAseq. **(B)** Correlation matrix of overall transcriptomic profiles. **(C, D)** Analysis of differentially expressed genes. Venn diagram **(C)** illustrating the strategy used to extract the common transcriptomic signatures **(D)** of sensitive and non-sensitive MCA-induced primary tumor cell lines.

In unsupervised principal component analysis (PCA) and correlation analyses on transcriptional profiles, all the tumors derived from the same parental primary cell line clustered together robustly **(** **Figures 6A, B** **)**. Despite the potentially significant clonal heterogeneity of the primary tumor cell lines, this excluded the possibility of a bias in our analyses due to heterogeneous growth upon transplantation. Furthermore, sensitive and non-sensitive tumors robustly segregated along the PC3 axis in PCA, with this axis itself accounting for a large proportion of the total variability between cell lines (21%) **(** **Figure 6A** **)**. Thus, the tumors in each of these groups had genetic determinants in common that were associated with their differential sensitivity.

We then sought to identify the transcriptional signature distinguishing between sensitive and non-sensitive tumors, by performing a global differential gene expression analysis. We first determined the specific signatures of each non-sensitive tumor relative to each sensitive tumor, and extracted their common determinants **(** **Figures 6C, D** **)**. This strategy identified 113 genes as differentially expressed between the two groups of tumors: 30 genes were more strongly expressed in sensitive than in non-sensitive tumors, and 83 genes were more strongly expressed in non-sensitive tumors **(** **Figure 6D** and **Table S1** **)**.

We focused our attention on transmembrane and secreted proteins, using automated GO annotations for each gene product, and examined their functions based on available reports **(** **Table S1** **)**. *Plet1* was the gene with the highest loading on the PC3 axis and was highly expressed in non-sensitive tumors. *Tnfsf11* was also upregulated in non-sensitive tumors. This gene encodes RANKL, for which induction and interaction with RANK have been associated with NK cell inhibition (20). The other genes upregulated in non-sensitive tumors included *Rasa4*, which controls RANKL expression; *Ifitm1*, which prevents NK cell cytotoxic activity in human gastric cancer; and *Vsir*, encoding the inhibitory immune checkpoint VISTA, which is homologous to PD-L1 (21–23). Conversely, several of the genes upregulated in sensitive tumors are involved in cell-cell interactions, including four members of the protocadherin family (*Pcdh*), the receptor neurotrimin (*Ntm*), and the kinase MUSK. The role of these genes in modulating NK cell/ILC1 functions in antitumoral responses is unclear and will be investigated in a follow-up study.

## Discussion

NK cells have been reported to inhibit tumor growth in transplantable tumor models (24). Their role during the development of primary tumors is less well documented. Studies in antibody-mediated depletion models have shown that NK cells and NKT cells prevent the development of primary tumors upon MCA injection (12). Consistent with this observation, we showed that tumor incidence after MCA injection was higher in mice treated with anti-NK1.1 depleting antibody than in control mice. Nevertheless, the depletion induced by this antibody is not fully specific to NK cells and/or ILC1, as it also targets NKT cells. We then used a mouse model with a specific deficiency of NKp46^+^ cells to investigate further. We found that NKp46^+^ cell deficiency did not alter the time-to-onset, growth rate or incidence of tumors after MCA injection. Overall, these results suggest that NK cells and ILC1 were dispensable for tumor immunosurveillance in the model of MCA-induced carcinogenesis, but provide support for a protective role of NKT cells in this process, as previously described (12). A study in another NKp46^+^ cell-deficient mouse model reported a higher incidence of tumors in deficient mice after the injection of MCA (19). Primary tumorigenesis in response to MCA is known to generate discordant results. The dose of MCA, the site of injection and the mouse strain used may underlie these discrepancies (25). However, conflicting results have been reported even for studies in which all these parameters were identical (2, 26). This observation suggests that other extrinsic factors, such as the mouse microbiota, may affect the outcome of primary carcinogenesis. Primary tumorigenesis experiments performed on both mouse strains in parallel are therefore required to reconcile these contradictory results concerning the role of NKp46^+^ cells in tumor immunosurveillance.

This carcinogenesis experiment led to the constitution of a library of 25 primary tumor cell lines. We found that 15 of these tumor cell lines grew slower in WT mice than in NK1.1^+^ cell-depleted mice. We investigated the cells responsible for the NK1.1^+^ cell sensitivity of the tumors, by analyzing the composition of tumor infiltrates. Based on the observation of the importance of NKT cells for tumor immunosurveillance, we initially focused on this population. Interestingly, we found that, after transplantation, there was a weak infiltration of NKT cells into the MCA-induced tumors, arguing against a role for these cells. Conversely, these tumors displayed high levels of infiltration with NKp46^+^ cells. These results suggest that, despite the dispensable nature of NKp46^+^ cells in the prevention of primary tumor development, these cells may play an active role in the antitumoral immune response established in transplantable tumor models, highlighting that distinct types of immune responses are acting in those two models. We therefore explored the mechanisms underlying the tumor sensitivity mediated by these cells.

Consistent with previous publications (19), the MCA-induced primary tumor cell lines contained three subpopulations of tumor-infiltrating NKp46^+^ cells: NK cells (CD49a^-^CD49b^+^), ILC1 (CD49a^+^CD49b^-^) and an intermediate population, int-ILC1, displaying characteristics of both populations (CD49a^+^CD49b^+^). It has been shown that NK cells can be converted into int-ILC1 through the action of TGF-β (19, 27). We therefore wondered whether tumor sensitivity stemmed from a decrease in the number of tumor-infiltrating NK cells due to their conversion into int-ILC1 and ILC1. However, the frequency of intratumoral NK cells was not correlated with NK1.1^+^ cell-dependent tumor growth.

We then investigated the possible contribution to tumor sensitivity of a differential capacity of the tumor cells to activate NK cells. However, we found that NK cells were able to kill MCA-induced primary tumor cell lines *in vitro* regardless of their sensitivity status. Our results therefore indicate that all MCA-induced tumor cell lines can activate NK cells directly, but that another process may occur in non-sensitive tumor beds, leading to the inhibition of intratumoral NK cells. Thorough testing of this hypothesis is required, but our RNAseq analysis of the tumor cells supports the hypothesis that the non-sensitive tumors have a more immunosuppressive microenvironment than sensitive tumors. Indeed, the genes upregulated in the non-sensitive tumors included *Vsir* (VISTA), *Plet1* (PLET1) and *Tnfsf11* (RANKL). VISTA is an inhibitory checkpoint displaying some sequence identity to PD-L1 (23). In human ovarian cancer, VISTA is expressed by tumor cells and impairs T cell-mediated antitumoral responses (28). In the AOM/DSS colon cancer model, PLET1 has been shown to be an IL-17A-induced protein (29). IL-17A is a protumoral cytokine promoting the recruitment of myeloid-derived suppressor cells (MDSCs) to the tumor bed (30). In human acute myeloid leukemia, activation of RANKL expressed by tumor cells leads to a dual inhibition of NK cells, through the secretion of immunomodulatory factors directly inhibiting NK cells and the upregulation of RANK expression by NK cells. RANKL-RANK interaction transduces the inhibitory signals in NK cells (20). The importance of RANKL in the mechanism of tumor sensitivity is supported by the associated upregulation of RASA4 expression in non-sensitive tumors, as RASA4 controls RANKL expression by regulating its shedding (21).

An analysis of the genes upregulated in sensitive tumors identified no genes known to be associated with activation of the antitumoral immune response. Nevertheless, protocadherins (*Pcdh*), a family of cell adhesion molecules, were well represented among these upregulated genes. Protocadherins are silenced by methylation in many human tumors and have been proposed as diagnostic biomarkers for cancer (31). In particular, protocadherin17 (*Pcdh17*) has been described as a tumor suppressor interfering with Wnt signaling (32). In our model, protocadherins acted more as a biomarker of the responsiveness of tumor cells to the antitumoral action of NK1.1^+^ cells than as a biomarker of tumorigenesis.

We also observed a weak, but significant inverse correlation between the frequency of tumor-infiltrating CD4^+^ T cells and the sensitivity status of the tumors. There is growing evidence to suggest that CD4^+^ T cells play an important role in the antitumor immune response. These cells can display cytotoxicity against tumor cells (33). They also provide help signals for the activation of cytotoxic CD8^+^ T cells (34). The non-sensitive tumors had higher levels of CD4^+^ T-cell infiltration than the sensitive tumors, suggesting that CD4^+^ T cell tumor infiltration may be a compensatory antitumor mechanism in non-sensitive tumors. However, it remains unclear whether the higher degree of CD4^+^ T cell infiltration was a cause or a consequence of alterations to the NK cell-mediated antitumor response.

The antitumoral activity of NK cells has been described in detail, but little is known about the involvement of ILC1 in the immune response in the tumor context. In human gastrointestinal cancers, ILC1 have been shown to be more abundant within tumors than in adjacent healthy tissues, but their role remains unclear (35). Our correlation analysis showed tumors with higher levels of ILC1 infiltration were less sensitive to NK1.1^+^ cells. Furthermore, we showed in the colon carcinoma MC38 tumor model that the depletion of NK1.1^+^ cells was protective in tumors with high levels of ILC1 infiltration. These results suggest that intratumoral ILC1 may have protumoral features. Several hypotheses can be formulated about the mechanisms underlying this observation. ILC1 are major producers of IFN-γ and TNFα. The tumor-promoting effects of these cytokines are now well known (19, 36). They may contribute to the generation of an immunosuppressive microenvironment by regulating the accumulation and functions of T regulatory (T_reg_) cells (37, 38) or MDSCs (39, 40). IFN-γ can also upregulate immune inhibitory checkpoint expression on tumor cells (41). Moreover, the tumor-promoting effects of ILC1 may result from the regulation of neo-angiogenesis. Tumorigenesis is dependent on vascular architecture, which supplies tumor cells with the nutrients and oxygen they require. It has been shown that NKp46^+^ cells, including ILC1 in particular, can promote tumor growth by stimulating the formation of an optimal network of blood vessels (19, 42). Furthermore, given that intratumoral ILC1 frequency is inversely correlated with the sensitivity of the tumor to NK1.1^+^ cells, we can hypothesize that ILC1 directly or indirectly inhibit the antitumor response mediated by NK cells. Additional experiments will be required to investigate these hypotheses and firmly characterize the role of ILC1 during anti-tumoral response. For this purpose, the generation of a specific and complete mouse model of selective deficiency of ILC1 would be important to decipher the cellular and molecular basis of their inhibitory function.

Overall, our results highlight an important link between tumor-infiltrating ILC1 and the regulation of tumor growth. Further, they support the hypothesis that low levels of sensitivity to NK1.1^+^ cells could not be directly link to a paucity of NK cells resulting from their conversion into non-cytotoxic ILC1. Rather, ILC1 would play an active role in inhibiting the antitumoral immune response.

## Methods

### Mice

C57BL/6 mice were purchased from Janvier. *Ncr1^Cre^R26^DTA^* were bred in-house (43). All mice were in specific pathogen–free conditions at the Centre d’Immunologie de Marseille-Luminy and the Centre d’Immunophénomique (CIPHE). Experiments were conducted in accordance with institutional guidelines for animal care and use.

### Carcinogenesis

Freshly prepared MCA (Sigma), diluted to the appropriate concentration in peanut oil (Sigma), was injected subcutaneously into the right flank of the mice. Tumor growth was measured weekly for 200 days with a caliper and expressed as a volume. Tumors were defined as palpable growing masses of more than 100 mm^3^ in volume. If required, the mice were treated with intravenous injections of 50 µg anti-NK1.1 antibody (clone PK136, BioXCell) every two weeks for 200 days.

### Primary Tumor Cell Lines and Tumor Transplantation

MCA-induced tumors were resected, minced, digested with collagenase IV, and passaged *in vitro* for two weeks. Multiple vials of low-passage number cells were then frozen. Cell lines were maintained in DMEM (Gibco^®^, Thermo Fisher Scientific) supplemented with 10% FCS (Gibco^®^, Thermo Fisher Scientific), as previously described. For transplantation experiments, aliquots were thawed, expanded, and 5.10^5^ cells were subcutaneously injected into the right flank of the mice. If required, mice were treated with intravenous injections of 50 µg anti-NK1.1 antibody (clone PK136, BioXCell) on the day before the injection of tumor cells and 15 days later. Tumor growth was measured every 2-3 days for 40 days with a caliper, and is expressed as a volume. The survival endpoint was set at 1,000 mm^3^. Tumors were resected, minced and single-cell suspensions were obtained with the Tumor Dissociation Kit and gentleMACS Octo Dissociator (Miltenyi Biotec), according to the manufacturer’s instructions.

### *In Vitro* Assays

Mice were primed with 100 µg poly(I:C) (*Invivo*gen) 24 hours before the experiments. Single-cell suspensions were obtained by mashing spleens through a strainer with 70 µm pores. Red blood cells were lysed with RBC lysis buffer (eBiosciences, Thermo Fisher Scientific). For CD107a/IFN-γ activation tests, splenocytes were cocultured with target cells for four hours in the presence of Golgi Stop™ and Golgi Plug™ protein transport inhibitors (BD Biosciences). For cytotoxicity assays, NK cell-enriched preparations were obtained from splenocytes with the NK Cell Isolation Kit (Miltenyi Biotec), according to the manufacturer’s instructions. These cells were then cocultured with^51^ Cr-labeled target cells for four hours.

### Flow Cytometry

Single-cell suspensions prepared from the indicated organs were incubated for 15 min at 4°C in 5 mM EDTA and 10 µg/mL of mouse Fc Block™ (rat anti-mouse CD16/CD32, BD Biosciences) in PBS, and were then stained by incubation for 20 min at 37°C with fluorochrome-conjugated mAbs. The following anti-mouse antibodies were purchased from BD Biosciences: IFNγ-AlexaFluor647 (clone XMG1.2), CD107α-FITC (clone 1D4B), CD45.2-AlexaFluor700 (clone 104), CD19-APC-Cy7 (clone 1D3), NKp46-BV421 (clone 29A1.4), NK1.1-BV510 (clone PK136), CD11b-BV650 (clone M1/70), TCRβ-BV711 (clone H57-597), CD49a-BV785 (clone Ha31/8), CD49b-BUV395 (clone HMa2), CD4-BUV737 (clone GK1.5). The tetramer CD1d-PBS57-PE was obtained by the NIH tetramer core facility. Dead cells were excluded from analyses by the electronic gating of cells negative for staining with fixable viability dye (Invitrogen). Data were collected on a FACS Canto or LSR-II flow cytometer (BD Biosciences) and analyzed with FlowJo software. After exclusion of doublets and dead cells, CD45-positive cells were gated. Within the CD45^+^ gate, B cells were defined as CD11b^-^CD19^+^ cells; NKT cells were defined as CD11b^-^CD19^-^TCRb^+^NK1.1^+^ or CD45^+^CD11b^-^CD19^-^TCRb^+^TetCD1d-PBS57^+^; CD8^+^ T cells were defined as CD11b^-^CD19^-^TCRb^+^NK1.1^-^CD8^+^CD4^-^; CD4^+^ T cells were defined as CD11b^-^CD19^-^TCRb^+^NK1.1^-^CD8^-^CD4^+^; NKp46^+^ cells were defined as CD11b^int/-^CD19^-^TCRb^-^NKp46^+^NK1.1^+^. Within the NKp46^+^ cells, NK cells were defined as CD49b^+^CD49a^-^; int-ILC1 were defined as CD49b^+^CD49a^+^ and ILC1 were defined as CD49b^-^CD49a^+^.

### RNAseq

Hematopoietic cells were removed from single-tumor cell suspensions with CD45 Microbeads and an autoMACS Pro Separator (Miltenyi Biotec), in accordance with the manufacturer’s instructions. CD45^-^ tumor cells were then frozen in RLT buffer (Qiagen) supplemented with 10 µL/mL 2-mercaptoethanol (Sigma). RNA extraction, library preparation and sequencing were performed by HalioDX (Marseille, France). For data analysis, fastq files were assessed with the fastqc program. Alignments were then generated with STAR 2.5.3a and the GRCm38 genome. The number of reads mapped to each gene was determined with featureCounts 1.6. Normalization and differential analysis were performed with the DESeq2 v1.26 R package.

## Data Availability Statement

The datasets presented in this study can be found in online repositories. The names of the repository/repositories and accession number(s) can be found below: https://www.ncbi.nlm.nih.gov/geo/GSE179753.

## Ethics Statement

The animal study was reviewed and approved by the Comité d’éthique en expérimentation animale de Marseille Ministère de l’enseignement supérieur, de la recherche et de l’innovation. Written informed consent was obtained from the owners for the participation of their animals in this study.

## Author Contributions

YK and EV initiated and designed the research. YK and MV wrote the manuscript, with the help of other coauthors. MV, ME, BE, JG, LS, SG, AF, and YK performed the experiments and/or analyzed and/or interpreted results. All authors contributed to the article and approved the submitted version.

## Funding

The EV laboratory at CIML and *Assistance-Publique des Hôpitaux de Marseille* is supported by funding from the European Research Council (ERC) under the European Union’s Horizon 2020 research and innovation program (TILC, grant agreement No. 694502 and MInfla-TILC, grant agreement No. 875102 - MInfla-Tilc), the *Agence Nationale de la Recherche* including the PIONEER Project (ANR-17-RHUS-0007), MSDAvenir, Innate Pharma and institutional grants to the CIML (INSERM, CNRS, and Aix-Marseille University) and to Marseille Immunopole. The funder was not involved in the study design, collection, analysis, interpretation of data, the writing of this article or the decision to submit it for publication.

## Conflict of Interest

AF and EV are employees of Innate Pharma.

The remaining authors declare that the research was conducted in the absence of any commercial or financial relationships that could be construed as a potential conflict of interest.

## Publisher’s Note

All claims expressed in this article are solely those of the authors and do not necessarily represent those of their affiliated organizations, or those of the publisher, the editors and the reviewers. Any product that may be evaluated in this article, or claim that may be made by its manufacturer, is not guaranteed or endorsed by the publisher.

## Supplementary Material

Supplementary Figure 1 The specificity of NKp46^+^ cell deficiency models. **(A)** Blood analysis of the *Ncr1^Cre/+^R26^+/+^* and *Ncr1^Cre/+^R26^DTA/+^* mice used in **Figure 1A** (100 µg MCA) (*n* = 24 mice per group). **(B)** Liver analysis of C57BL/6 mice 7 days after the intravenous injection of anti-NK1.1 antibody (*n* ≥ 7 mice per group). (Welch’s *t* tests; ***p *< 0.01, ****p *< 0.005).

Supplementary Figure 2 Survival curves for each of the MCA-induced primary tumor cell lines tested. Groups of 5 C57BL/6 mice were transplanted subcutaneously with MCA-induced primary tumor cell lines, with and without anti-NK1.1 depleting antibody treatment. Tumors were classified as sensitive or non-sensitive on the basis of the *p*-values of the LogRank tests for the survival curves.

Supplementary Figure 3 Infiltration of NKp46^+^ NK1.1^+^ cell subpopulations in other tumor models. B16F10 melanoma **(A)** and MC38 adenocarcinoma **(B, C)** cell lines were subcutaneously injected into the indicated mice. Mice were killed and the tumor-infiltrating NKp46^+^ NK1.1^+^ cell subpopulations were characterized when the tumors reached a volume of 1,000 mm^3^.

Supplementary Table 1 Genes differentially expressed between sensitive and non-sensitive tumors. List of the genes differentially expressed, as illustrated by the heatmap in **Figure 6D**. The genes identified as secreted or associated with the plasma membrane by automated GO annotation are highlighted in bold typeface.
