# Supplementary material for: Type 1 Innate Lymphoid Cells Limit the Antitumoral Immune Response
Source: Front Immunol. 2021 Nov 16;12:768989. doi: 10.3389/fimmu.2021.768989 (PMC8637113; doi:10.3389/fimmu.2021.768989)
Supplement: Supplementary Figure 1 — The specificity of NKp46+ cell deficiency models. (A) Blood analysis of the Ncr1Cre/+R26+/+ and Ncr1Cre/+R26DTA/+ mice used in Figure 1A (100 µg MCA) (n = 24 mice per group). (B) Liver analysis of C57BL/6 mice 7 days after the intravenous injection of anti-NK1.1 antibody (n ≥ 7 mice per group). (Welch’s t tests; **p < 0.01, ***p < 0.005). [file DataSheet_1.docx]

**Type 1 innate lymphoid cells limit the antitumoral immune response**

Margaux Vienne^1^, Marion Etiennot^1^, Bertrand Escalière^1^, Justine Galluso^1^, Lionel Spinelli^1^, Sophie Guia^1^, Aurore Fenis^3^, Eric Vivier^1,2,3*^, Yann M. Kerdiles^1,*^

**Supplementary materials**


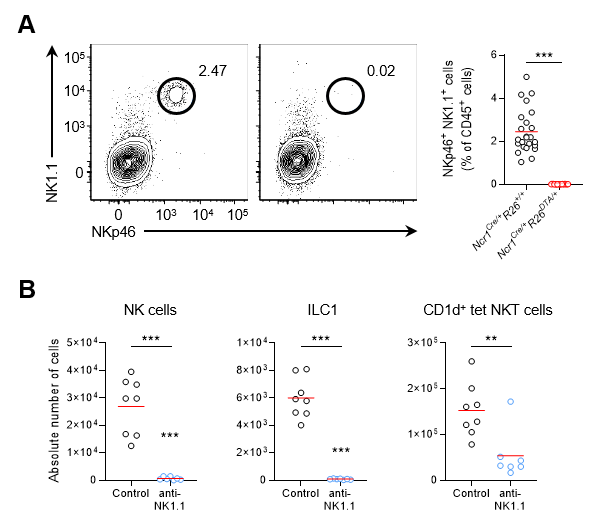


**Figure S1. The specificity of NKp46^+^ cell deficiency models**

(**A**) Blood analysis of the *Ncr1^Cre/+^R26^+/+^* and *Ncr1^Cre/+^R26^DTA/+^* mice used in Figure 1A (100 µg MCA) (*n* = 24 mice per group). (**B**) Liver analysis of C57BL/6 mice 7 days after the intravenous injection of anti-NK1.1 antibody (*n* ≥ 7 mice per group). (Welch’s *t* tests; ***p*<0.01, ****p*<0.005).


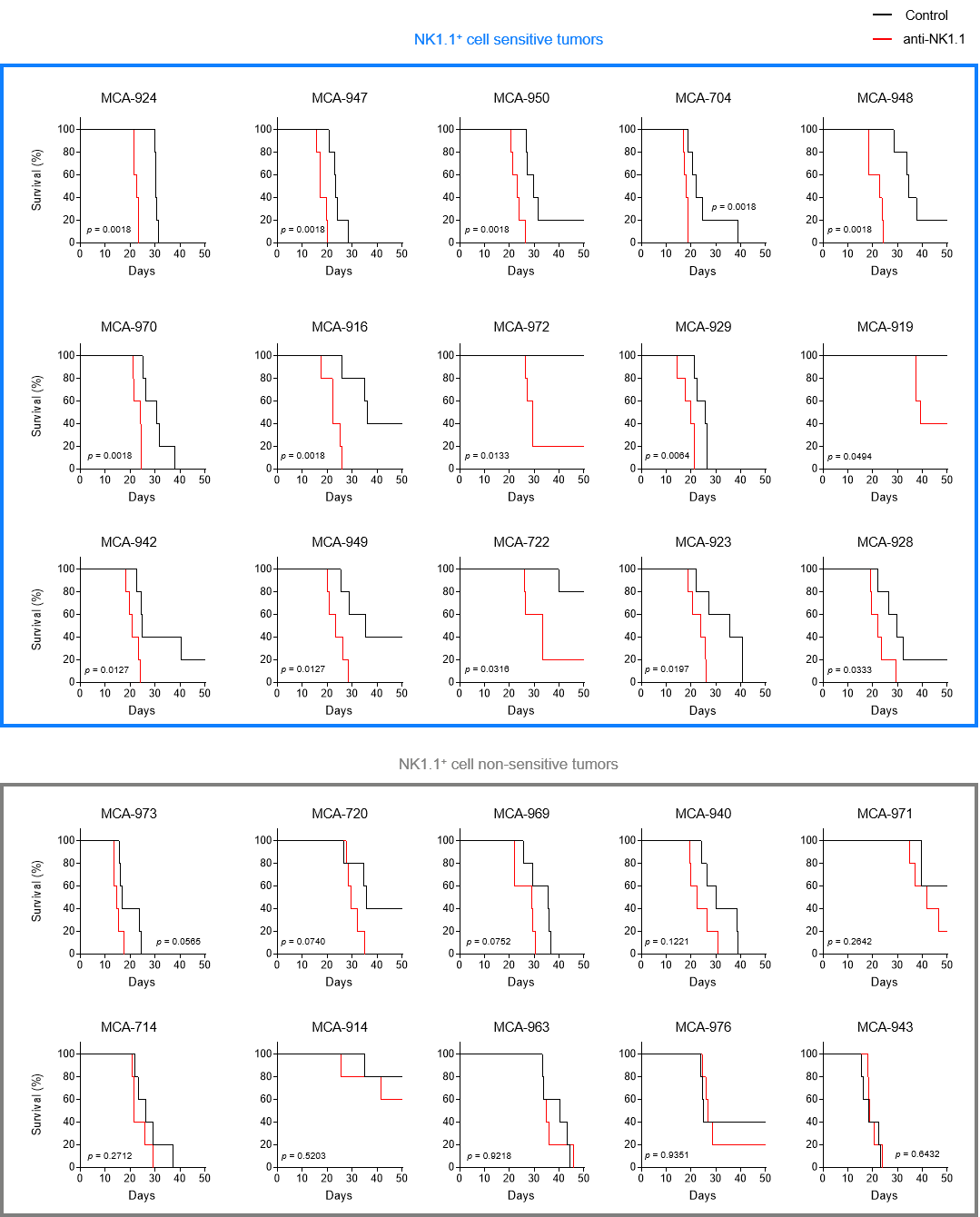


**Figure S2. Survival curves for each of the MCA-induced primary tumor cell lines tested**

Groups of 5 C57BL/6 mice were transplanted subcutaneously with MCA-induced primary tumor cell lines, with and without anti-NK1.1 depleting antibody treatment. Tumors were classified as sensitive or non-sensitive on the basis of the *p*-values of the log-rank tests for the survival curves.


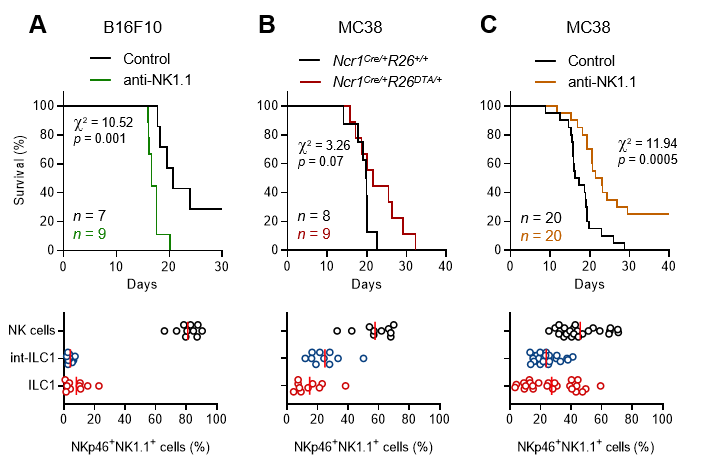


**Figure S3. Infiltration of NKp46^+^NK1.1^+^ cell subpopulations in other tumor models**

B16F10 melanoma (**A**) and MC38 adenocarcinoma (**B, C**) cell lines were subcutaneously injected into the indicated mice. Mice were killed and the tumor-infiltrating NKp46^+^NK1.1^+^ cell subpopulations were characterized when the tumors reached a volume of 1,000 mm^3^.

| **Genes upregulated in non-sensitive tumors** | | | | | | | | |
| --- | --- | --- | --- | --- | --- | --- | --- | --- |
| Trbc2 | | **Plet1** | **Pcsk9** | Hoxa7 | Cyp27a1 | Rassf6 | **Tnfsf11** | |
| **Cdh3** | | Sh3bp5 | **Sla2** | **Car11** | Trbj2-3 | Fbxo27 | Ptprn2 | |
| Trbj2-7 | | **Nipsnap1** | Hmgcll1 | Dlx2 | Gm15767 | Nuggc | Gm44905 | |
| Pdzrn4 | | Gm10421 | **Lgals7** | **Drd4** | Fahd2a | **Slc16a4** | C430042M11Rik | |
| **Grik5** | | Klhdc8a | Scml4 | **Rasa4** | Rgs11 | **Cntnap1** | 6330403K07Rik | |
| Sh3gl3 | | Fam161a | Lrrn4cl | Zfp991 | **Ezr** | Renbp | 1700001O22Rik | |
| Sncg | | **Ifitm1** | B4galnt1 | **Erbb3** | Crabp2 | **Wnt6** | 9030617O03Rik | |
| **Fxyd1** | | Eps8l2 | Eps8l1 | **Itgb4** | Gm14137 | **Vsir** | 2310030G06Rik | |
| **Col28a1** | | **Palm3** | Acsm3 | Zfp296 | **Camk2a** | Zfp784 | Mrpl23-ps1 | |
| **Acy3** | | **Sfn** | **Hc** | Cryaa | **Gpr68** | **Fgf2** | Mapkapk3 | |
| **Stxbp2** | | **Trpm6** | Jup | Ckb | Cdk18 |  |  | |
|  |  | |  |  |  |  |  | |
|  |  | |  |  |  |  |  | |
| **Genes downregulated in non-sensitive tumors** | | | | | | | | |
| Ndn | **Pcdhgb2** | | **Musk** | Srgap1 | **Pcdhgb7** | **Pag1** | Foxf2 | **Art3** |
| **Diras2** | **Fam189a1** | | **Arrdc4** | **Adcy2** | **Pcdh17** | Spaca1 | **Cnksr2** | **Ptx3** |
| **Ntm** | **Pcdhga5** | | Lonrf3 | Elavl2 | Gm15473 | **Adamts3** | 1700001L05Rik | |
| Cln8 | Tbc1d9 | | Eya1 | **Lypd6** | **Slc7a11** | Bend7 | 9030624G23Rik | |

**Table S1. Genes differentially expressed between sensitive and non-sensitive tumors**

List of the genes differentially expressed, as illustrated by the heatmap in Figure 6D. The genes identified as secreted or associated with the plasma membrane by automated GO annotation are highlighted in bold typeface.
